# Single and Combined Mutations of Acetylcholinesterase Gene Giving Resistance to Pirimiphos-Methyl in *Musca domestica* Slaughterhouse Populations

**DOI:** 10.3390/insects14030218

**Published:** 2023-02-22

**Authors:** Ali A. Alzabib, Ali S. Al-Sarar, Yasser Abobakr, Amgad A. Saleh

**Affiliations:** 1 Department of Plant Protection, College of Food and Agriculture Sciences, King Saud University, P.O. Box 2460, Riyadh 11451, Saudi Arabia; 2 Department of Animal Pests, Plant Protection Research Institute (PPRI), Agricultural Research Center (ARC), Alexandria 21616, Egypt; 3 Agricultural Genetic Engineering Research Institute (AGERI), Agriculture Research Center (ARC), Giza 12619, Egypt

**Keywords:** pirimiphos-methyl, *Musca domestica*, acetylcholinesterase, Saudi Arabia, resistance, mutation

## Abstract

**Simple Summary:**

The house fly is a worldwide public health pest associated with humans and livestock. The use of insecticides is still the main method of house fly management. The extensive use of insecticides is the main reason for the development of resistance in many insect species. The development of resistance in house flies was documented for numerous insecticides. Pirimiphos-methyl is used for house fly control in Saudi Arabia. This study was conducted to evaluate the resistance of *M. domestica* field populations against pirimiphos-methyl. The field-collected populations of *M. domestica* displayed different levels of resistance. The samples collected from Riyadh city exhibited the highest resistance, followed by populations from Jeddah and Taif. We investigated the genetic mutations of the acetylcholinesterase (*Ace*) gene in field-collected flies and the survivors after exposure to pirimiphos-methyl. The outcomes of the present study provide valuable information that may help in house fly management in Saudi Arabia.

**Abstract:**

The house fly *Musca domestica* L. (Diptera: Muscidae) is a worldwide medical and veterinary pest, causing great economic losses. Organophosphate insecticides have been widely used to control house fly populations. The main objectives of the present study were to evaluate the resistance levels of *M. domestica* slaughterhouse populations, collected from Riyadh, Jeddah, and Taif, against the organophosphate insecticide pirimiphos-methyl and investigate the genetic mutations of the *Ace* gene associated with pirimiphos-methyl resistance. The obtained data showed that there were significant differences among pirimiphos-methyl LC_50_ values of the studied populations, where the highest LC_50_ was recorded for the Riyadh population (8.44 mM), followed by Jeddah and Taif populations (2.45 mM and 1.63 mM, respectively). Seven nonsynonymous SNPs were detected in the studied house flies. The Ile239Val and Glu243Lys mutations are reported for the first time, whereas Val260Leu, Ala316Ser, Gly342Ala, Gly342Val, and Phe407Tyr were previously reported in *M. domestica* field populations from other countries. Considering three mutations associated with insecticide resistance, at amino acid positions 260, 342, and 407 of acetylcholinesterase polypeptide, 17 combinations were recovered in this study. Three out of these seventeen combinations were frequently found both worldwide and in the three Saudi house fly field populations, as well as their pirimiphos-methyl-surviving flies. Overall, the single and combined *Ace* mutations are apparently associated with pirimiphos-methyl resistance, and the obtained data can be useful in managing house fly field populations in Saudi Arabia.

## 1. Introduction

The house fly *Musca domestica* (Diptera: Muscidae) is a worldwide medical and veterinary pest. It is often found in areas of human activities, e.g., houses, workplaces, food markets, restaurants, slaughterhouses, animal and poultry farms. These places are suitable for *M. domestica* reproduction due to the availability of organic materials [1]. In addition to being a nuisance pest, house flies are responsible for the transmission of more than 200 pathogens, including bacteria, viruses, fungi, and parasites, which cause serious diseases in humans and animals [2,3].

House fly populations can be controlled using a variety of strategies, including environmental sanitation, chemical, physical, and mechanical control. Insecticide chemicals, which come in a variety of formulations and treatment techniques, are the greatest options for quick, inexpensive, and practical house fly management. In several countries, organophosphate (OP) insecticides have been used extensively to control house flies [4,5,6]. In Saudi Arabia, the extensive use of insecticides for a long time has led to the development of resistance in house fly populations [7,8,9,10].

Resistance to OP insecticides has been linked to metabolic resistance via the increased activity of detoxifying enzymes and/or acetylcholinesterase (AChE) insensitivity [6,11]. Carbamate and OP insecticides are structural analogs of acetylcholine, and they can inactivate the insect AChE enzyme, leading to an excess of acetylcholine at cholinergic synapses, eventually causing paralysis and death of insects [12,13]. To overcome the sensitivity of AChE to OP and carbamate insecticides, house flies develop resistance by creating mutations in the acetylcholinesterase (*Ace*) gene that produces AChE insensitivity to insecticides [6,14,15,16]. Currently, seven nonsynonymous mutations (A/G-495 ˃ Ile162Met, G/C-787 ˃ Val260Leu, C/T-929 ˃ Thr310Met, G/T-946 ˃ Ala316Ser, G/C/T-1025 ˃ Gly342Ala/Val, T/A-1220 ˃ Phe407Tyr, and G/C-1334 ˃ Gly445Ala), existing singly or in combination and associated with OPs resistance, have been identified in the house fly *Ace* gene [5,15,17,18]. The emergence of resistance may lead to an increase in the quantity and frequency of insecticide applications in both domestic and commercial livestock and poultry farms, raising the expense of control and having detrimental environmental effects [19,20]. The main objectives of the present study are to (1) evaluate the resistance levels of *M. domestica* slaughterhouse populations, collected from Riyadh, Jeddah, and Taif, against OP insecticide pirimiphos-methyl (PM); and (2) investigate the genetic mutations of the *Ace* gene in collected field flies and their counterpart survivors after exposure to PM. The outcomes of the present study provide valuable information that may help in controlling house fly populations in Saudi Arabia.

## 2. Materials and Methods

### 2.1. Collection and Rearing of House Flies

Field populations of house flies were collected by sweep net from slaughterhouses (about 100 adult house flies/population) in different regions of Saudi Arabia, namely Riyadh (Riyadh Automated Slaughterhouse, N24.5793252166357, E46.73542482697681), Jeddah (East Jeddah Slaughterhouse, N21.53832286527901, E39.25652725404367), and Taif (Taif Municipality Ideal Slaughterhouse, N21.34799321587203, E40.45159577841662). The collected house flies (either live or preserved in 95% ethanol) were transported to the Insect Breeding Laboratory, Department of Plant Protection, College of Food and Agriculture Sciences, King Saud University. The house flies stored in 95% ethanol were used for molecular analysis.

The live house flies were designated as the parental generation and were allowed to randomly mate. The F1 progeny (3–5-days-old) were used for toxicity bioassays. The susceptible laboratory strain (LAB, bred since 2006) was brought from the Public Health Pests Laboratory (PHLP-Jeddah, Jeddah, Saudi Arabia). The LAB strain and field house flies were kept at 25 ± 2 °C, 30–40% relative humidity, and in a 12:12 h light-dark cycle. Adult house flies were fed on a mixture of 2% milk powder and 10% sucrose. Eggs were collected from the cages and cultured in a larval medium containing wheat bran, yeast, milk powder, and water at the proportions of 20:1:2:20, respectively [21].

### 2.2. Insecticide

The OP insecticide pirimiphos-methyl (*O*-[2-(Diethylamino)-6-methylpyrimidin-4-yl] *O*,*O*-diethyl phosphorothioate, 90.5%) (Hunan Haili Chemical Industry Co., Hannan, China) was used in this study.

### 2.3. Bioassays

The topical application method was used for assessing the resistance levels in the house flies towards PM, according to Scott et al. [22]. A 32.75 mM stock solution of 90.5% PM was first prepared in acetone and then diluted to at least 5 serially diluted concentrations. Based on the preliminary dose–response, the 5 PM concentrations for the LAB strain, Riyadh, Jeddah, and Taif were 0.0033, 0.0066, 0.013, 0.026, and 0.052 mM; 3.28, 6.55, 9.83, 13.1, and 16.4 mM; 1.64, 2.46, 3.28, 4.9, and 6.55 mM; and 0.33, 0.66, 1.31, 2.62, and 5.24 mM, respectively. An amount of 1 µL of each diluted insecticide concentration was applied on the thoracic notum of 3–5-day-old flies. For control treatments, 1 µL of acetone was applied for each fly. Four replicates were used for each concentration and each replicate had twenty flies. Flies were anesthetized by CO_2_ for 20 s before their treatments. The treated flies were maintained in glass cups (250 mL) with cotton inside that had been wet with a 20% sugar solution at 25 ± 2 °C, under a 12:12 h light-dark cycle. Mortality was assessed 24 h after treatment. Flies that were not moving when touched with a soft brush were scored as dead.

### 2.4. Ace Genotyping

Genomic DNA was extracted from individual house flies preserved in 95% ethanol using the QIAamp DNA Mini Kit (Qiagen, Hilden, Germany), according to the manufacturer’s protocols. The quality and quantity of DNA solutions were determined with a Nanodrop spectrophotometer (Thermo Scientific™, Waltham, MA, USA) and agarose gels, according to Sambrook and Russell [23]. For PCR reactions, the concentrations of extracted DNA solutions were adjusted to 20 ng/µL and then stored at −20 °C for further molecular work [23].

The *Ace* gene fragment was amplified by PCR in a 30 μL reaction system containing 15 μL of 2 × Go Taq Green Master mix (Promega Corporation, Madison, WI, USA), 10 μL of Nuclease-Free Water, 2 μL of DNA template, and 1.5 μL of each of 10 μM forward primer S90MdAce (5′-CATCTAAAACCGATCAGGACCATTTAATAC-3′) and 10 μM reverse primer AS89MdAce (5′-TCATCTTTAACATTTCCAATCAGAATATCG-3′) [16]. PCR conditions were as follows: 94 °C for 3 min, followed by 35 cycles of 94 °C for 30 s, 55 °C for 30 s, and 72 °C for 60 s, and a final extension step of 5 min at 72 °C [16]. PCR products were run on 1.5% agarose gels stained with 0.5 µg/mL acridine orange. PCR products were directly sequenced at Macrogen sequence facility (Macrogen, Seoul, Korea). DNA sequences were cleaned and edited manually using BioEdit (https://archive.org/details/bioedit; accessed on 5 October 2021) and Geneious (https://www.geneious.com; accessed on 3 March 2022) software. The cleaned sequences were searched against the NCBI-GenBank database to get their homologous counterparts. BioEdit was used to detect single nucleotide polymorphisms (SNPs) and assign combinations of different mutations.

### 2.5. Statistical Analysis

The median lethal concentration (LC_50_) for PM was determined by probit analysis [24]. Significant differences between LC_50_ values were based on no overlapping, 95% confidence intervals [25]. Resistance ratios (RRs) were calculated by dividing LC_50_ of a field population by LC_50_ of the laboratory strain [26]. To assess the relationship between the frequency of single or combined mutations of the *Ace* gene and PM-RRs, Pearson correlation coefficient (*r*) was calculated. The analyses were conducted using SPSS 26 software (IBM, Armonk, NY, USA). In order to reveal possible genetic relationships among the three house fly field populations, the frequencies of *Ace* genotypes recovered from them were tested for conformity to Hardy–Weinberg equilibrium, using the Exact HWE test embedded in Genepop 4.7.5 [27]. In addition, pairwise F_ST_ estimates [28] and number of migrants (Nm) [29] were calculated with Genepop.

## 3. Results

### 3.1. Susceptibility of House Flies to PM

The LC_50_ values of adult *M. domestica* slaughterhouse populations, collected from Riyadh, Jeddah, and Taif regions in Saudi Arabia, were significantly higher than that of the LAB strain (Table 1). Moreover, there were significant differences among PM-LC_50_ values of the three slaughterhouse populations (Table 1). The highest LC_50_ was recorded for the Riyadh population (8.44 mM), followed by Jeddah and Taif populations, with LC_50_ values of 2.45 and 1.63 mM, respectively. The recorded RRs were 602.9, 175, and 116.4-fold for Riyadh, Jeddah, and Taif populations, respectively (Table 1).

### 3.2. Genotyping of Ace Resistance Alleles

An 842 bp fragment of the *Ace* gene was amplified by PCR from 172 individual house flies. The *Ace* fragment contained one 85 bp intron. A total of 16 SNPs were detected: 7/16 from the exon and 9/16 from the intron. The seven SNPs in exon region were nonsynonymous, causing amino acid changes in AChE protein. These mutations were Ile239Val, Val260Leu, Glu243Lys, Ala316Ser, Gly342Ala, Gly342Val, and Phe407Tyr (Table 2). Single, double, and triple peaks per SNP were detected in the *Ace* fragment (data not shown). There were six individuals that showed triple peaks at position G/C/T-1025: two from Taif and four from Jeddah slaughterhouse populations.

### 3.3. SNPs

The four mutations, namely Val260Leu, Gly342Ala, Gly342Val, and Phe407Tyr, of *Ace* gene, conferring resistance against OPs in insects, were detected in the Saudi house fly field populations (Table 2). In addition, three more mutations were detected: Ile239Val (found in Riyadh and Jeddah populations), Glu243Lys (found in the Riyadh population), and Ala316Ser (found in the Taif population) (Table 2). The two genotypes, 260 Val/Leu and 260 Leu/Leu, with differing frequencies were found in the Riyadh field population, whereas 260 Val/Val homozygote was not detected (Table 3). However, the three 260-genotypes were found in the Jeddah and Taif field populations. At the 342 position, three genotypes (342 Gly/Ala, 342 Ala/Ala, and 342 Ala/Val) were detected in the Riyadh population (Table 3). However, four (342 Gly/Gly, 342 Gly/Ala, 342 Ala/Ala, and 342 Ala/Val) and five genotypes (342 Gly/Gly, 342 Gly/Ala, 342 Gly/Val, 342 Ala/Ala, and 342 Ala/Val) were found in Jeddah and Taif field populations, respectively (Table 3). The three possible genotypes (407Phe/Tyr, 407Phe/Phe, and 407Tyr/Tyr), at the 407 position, were observed in the three house fly field populations (Table 3). For the survivors of Riyadh house flies exposed to 13.1–16.4 mM of PM, the percentages of mutated alleles at positions 260, 342, and 407 were 87.5%, 75%, and 58.3%, respectively (Table 2). For the survivors of Jeddah flies exposed to 4.9–6.55 mM of PM, the nonsynonymous mutations were detected at positions 239, 260, 342, and 407, with frequencies of 12.5%, 83.3%, 100%, and 91.7%, respectively (Table 2). The percentages of mutated alleles, at positions 260, 342, and 407, found in the survivors of Taif flies, treated with 2.62–5.24 mM of PM, were 66.7%, 88.9%, and 88.9%, respectively (Table 2). There were strong positive correlations of PM-RRs of Riyadh, Jeddah, and Taif house fly populations with Phe407Tyr (*r* = 0.997, *p* = 0.02), Gly342Ala (*r* = 0.99, *p* = 0.03), and Val260Leu (*r* = 0.95, *p* = 0.10) mutations; however, the latter correlation was not significant.

### 3.4. Ace Combinations

When the three *Ace* mutations at positions 260, 342, and 407 (associated with OP resistance) were considered together, nine different combinations were detected in the Riyadh population (Table 4). The Leu260 + Ala342 + Tyr407 combination was the most frequent one (22/48), followed by Val/Leu260 + Ala/Val342 + Tyr407 (7/48) (Table 4). Four combinations were represented by more than one individual fly, namely Val/Leu260 + Gly/Ala342 + Phe/Tyr407 (5/48), Leu260 + Ala342 + Phe/Tyr407 (5/48), Val/Leu260 + Gly/Ala342 + Phe407 (3/48), and Leu260 + Ala/Val342 + Tyr407 (3/48). The three remaining combinations were only represented by one individual (Table 4). The survivors in the Riyadh population showed six combinations, with only one combination (Leu260 + Gly/Ala342 + Phe/Tyr407) that was not detected in the field population (Table 4). In the Jeddah population, 10 different combinations were detected. As in the case of the Riyadh population, the most frequent combination was Leu260 + Ala342 + Tyr407 (15/47), followed by Leu260 + Ala342 + Phe/Tyr407 (9/47). Four combinations had more than individuals: Leu/Tyr260 + Ala/V342 + Phe/Tyr407 (7/47), Val/Leu260 + Ala/Val342 + Tyr407 (6/47), Val/Leu260 + Gly/Ala342 + Tyr407 (3/47), and Val/Leu260 + Gly/Ala/Val342 + Tyr407 (3/47). The other four combinations were represented by one individual fly each, while one combination, Val260 + Gly342 + Phe407, had three sensitive alleles (Table 4). The survivors in the Jeddah population showed three combinations that had already been detected in the field population (Table 4). The Taif population had 13 *Ace* combinations, 9 of which were represented by one individual each, with one sensitive combination (Table 4). The four multi-individual combinations were Val/Leu260 + Ala/Val342 + Tyr407 (11/44), Leu260 + Ala342 + Phe/Tyr407 (11/44), Val260 + Ala342 + Tyr407 (8/44), and Val/Leu260 + Ala/Val342 + Phe/Tyr407 (5/44). Three out of the four combinations recovered from survivors in the Taif population had already recovered from the field population. Interestingly, the fourth combination, Leu260 + Ala/Val342 + Tyr407 (represented by 33% of the survivors), was not detected in the field population. Overall, the most frequent combination, Leu260 + Ala342 + Tyr407, was found to be strongly, positively correlated with PM-RRs for the three populations (*r* = 0.92, *p* = 0.078).

### 3.5. Ace Genotypes Recovered from Field House Fly Populations and Their PM-Surviving Counterparts

When the whole *Ace* fragment was considered for analysis, 31 *Ace* genotypes were found among the 48 individual house flies of the Riyadh field population. Among the 31 *Ace* genotypes, there were 26 singletons and 5 multi-individual genotypes with 8, 3, 6, 2, and 3 individuals each (Table 5). An amount of 10 *Ace* genotypes were recovered from 12 individuals who survived F1 offspring flies from the Riyadh population exposed to PM. The 10 heterozygous genotypes included 8 singletons and 2 multi-individual genotypes, with 2 individuals each. Interestingly, there were 5 genotypes not recovered from the field population. The Jeddah population showed 33 *Ace* genotypes, that included 26 singletons and 7 multi-individual genotypes, with the following frequencies: 6, 4, 3, 2, 2, 2, and 2 each (Table 5). A total of 1 out of the 26 singleton genotypes had the susceptible *Ace* allele. Ten *Ace* genotypes (nine singletons and one genotype with two individuals) were recovered from the eleven survivors of Jeddah F1 flies exposed to PM. Among the 10 surviving *Ace* genotypes of Jeddah, 5 were not recovered from the field population. From the 44 house flies of the Taif population, 29 genotypes were found, with 23 singletons and 6 multi-individual genotypes with 2, 2, 2, 3, 3, and 8 individuals each (Table 5). One susceptible genotype was represented among the 23 singletons. A total of 8 *Ace* genotypes (5 were not recovered from the field population) were found among the 10 survivors of Taif F1, with 7 singletons; the eighth one had 3 individual flies (Table 5). Based on the F_is_ estimates, with probabilities very close to zero, the three field populations showed deviation from Hardy–Weinberg equilibrium (Table 5). The values of the pairwise F_ST_ estimates for the Ace genotypes of three housefly field populations were very low, along with high gene flow (Nm ≥ 8), indicating that there is little genetic differentiation among these populations (Table 6).

## 4. Discussion

The use of insecticides is still the primary means of controlling house flies in places such as animal production and slaughterhouses [5]. The extensive use of insecticides is the main reason for the development of resistance to these chemicals in various insect species [30]. The development of resistance in house flies was documented for numerous insecticides [5,16,31,32]. Pirimiphos-methyl is one of the common OP insecticides used for house fly control in Saudi Arabia [33]. This study was conducted to evaluate the resistance of *M. domestica* field populations, collected from slaughterhouses in three cities (Riyadh, Jeddah, and Taif), against PM. The field-collected populations of *M. domestica* displayed different levels of resistance to PM. The samples collected from Riyadh city exhibited the highest resistance, followed by populations from Jeddah and Taif, with RR values of 602.9, 175, and 116.4-fold, respectively. Several studies have documented house fly resistance to OP insecticides worldwide [5,34,35]; however, few studies documented *M. domestica* resistance to PM. Kočišová et al. [36] reported 40-fold increase in the house fly resistance to PM after exposing the flies to the insecticide for 10 weeks. The resistance to PM has also been found in other important insects, such as *Rhyzopertha dominica*, *Bemisia tabaci*, *Aedes aegypti*, and *Anopheles gambiae* [37,38,39,40,41]. In Saudi Arabia, OP insecticides have been used to control public health pests, and the development of house fly resistance has been reported [9,10,42,43]. House fly populations collected from Riyadh city showed an increase in the resistance rate over time to OPs [9,10]. For example, Abobakr et al. [10] reported higher RRs (62.47–309.78 folds) to diazinon compared with those reported earlier, by Alzahrani et al. [9], (6.8–72 folds) in field populations collected from the same slaughterhouses in Riyadh. Indeed, the use of insecticides with a long residual effect against flies, in closed facilities, can lead to a rapid development of resistance due to selection pressure, rapid replication rate, and absence of sensitive fly dilution effect [36].

Understanding the genetic basis of insecticide resistance in house fly field populations is critical to develop effective and successful control strategies. Many mutations detected in the *Ace* gene have been found to be associated with OP resistance in house flies (Table 7) [15,16,17,18,34,44]. However, three point mutations at *Ace* nt positions, G/C-787, G/C/T-1025, and T/A-1220, are considered the most important in offering OP resistance in house flies [15,16,17]. These point mutations lead to changes in the amino acid composition of the AChE enzyme as follows: G/C-787 ˃ Val260Leu, G/C/T-1025 ˃ Gly342Ala/Val, and T/A-1220 ˃ Phe407Tyr [15,16,17]. In this study, an 842 bp fragment of the *Ace* gene was amplified from individual house flies representing three field populations, and their survivors were exposed to PM-insecticide. Seven nonsynonymous SNPs (A/G-715 ˃ Ile239Val, G/A-727 ˃ Glu243Lys, G/C-787 ˃ Val260Leu, G/T946 ˃ Ala316Ser, G/C-1025 ˃ Gly342Ala, G/T-1025 ˃ Gly342Val, and T/A-1220 ˃ Phe407Tyr) were detected in the *Ace* fragment of studied flies. The detected SNPs were mostly represented by single peaks in the chromatograms. However, double and triple peaks in single positions were also detected. The double peaks indicate the presence of two alleles at a single nucleotide position (a heterozygous SNP). The triple peaks, detected at the G/C/T-1025 of *Ace* sequences, indicate more than two alleles at that position, suggesting the existence of more than one copy of *Ace* in the *M. domestica* genome. The triple peaks in house fly *Ace* sequences were previously detected in different populations, e.g., in the USA [5]. Many studies reported the existence of *Ace* gene duplication in mosquitoes, e.g., *Anopheles gambiae* and *Culex pipiens* [45,46,47]. Further studies are needed to confirm the existence of *Ace* gene duplication and to investigate their fitness cost, associated with OP resistance, in field populations of house flies.

The individual contribution of single amino acid changes, resulting from single *Ace* point mutations, can differ in their influence on the house fly insensitivity to OP insecticides [15,17]. Kozaki et al. [17] and Walsh et al. [15] reported that Val260Leu mutation in house fly AChE may confer relatively limited levels of insecticide insensitivity. In this study, there was a positive correlation between RRs and Val260Leu mutation; however, the correlation was not significant. For the Gly342Ala/Val mutation, Walsh et al. [15] showed that it resulted in much stronger resistance in house fly populations. The change of Gly to either Ala or Val, at the amino acid position 342, results in increasing the size of its side chains and decreasing the area of the active site of AChE [48]. The strong positive correlation between PM-RRs and Gly342Ala mutation (*r* = 0.99, *p* = 0.03) supports the previous findings. The replacement of Phe with Tyr, at 407 position of AChE polypeptide, showed a minor effect on its insensitivity [15]. In this study, the three amino acid replacements at positions 260, 342, and 407 were detected in both field populations and their PM-surviving counterparts. Moreover, wild type amino acids (Val260, Gly342, and Phe407) at the three positions were also detected in both field populations and their counterpart survivors, except the Jeddah survivors (Table 2). In addition, the homozygous genotypes of these wild type amino acids were detected in field populations, except for the Riyadh population, where Val/Val and Gly/Gly, at positions 260 and 342, respectively, were not detected (Table 3). As in other worldwide house fly populations, Val/Val homozygotes at the 342 position were not recovered [4,6,16,44]. Based on previous studies and this study, the three mutations at positions 260, 342, and 407 confer different levels of insecticide insensitivity in house flies [4,15,17,18,48,49].

When the three individual mutations (at amino acid positions 260, 342, and 407) are combined, they show a strong effect on AChE activity [15,17,18,49]. For example, 342Val mutation always co-occurs with the 407Tyr mutation in insecticide-resistant house flies [15,32,44]. In this study, seventeen combinations, four homologous and thirteen heterozygous, of Val260Leu, Gly342Ala/Val, and Phe407Tyr mutations were recovered. The sensitive wild type (Val + Gly + Phe) was one of four homologous combinations and was represented by only one individual fly in Jeddah and Taif field populations. The homozygous Leu + Ala + Tyr was the most frequently detected combination, with frequencies of 46%, 32%, and 19% in Riyadh, Jeddah, and Taif field populations, respectively. In addition, the same combination was recovered from PM-surviving flies. This most frequent combination was strongly, positively correlated with the corresponding PM-RR 602.9, 175, and 116.4 values for Riyadh, Jeddah, and Taif, respectively. Walsh et al. [15] reported that house fly strain 77M, possessing Leu + Ala + Tyr combination, showed 48-fold DDVP resistance compared with the wild type. This combination was also frequently detected in house fly field populations in the USA, Turkey, Japan, Kazakhstan, and China [4,6,16,17,35,48]. Among the 13 heterogeneous combinations, Val/Leu + Ala/Val + Tyr and Leu + Ala + Phe/Tyr were well represented in the surveyed regions. Although these two combinations were recovered from the PM-survivors of Riyadh and Jeddah flies, they were not recovered from the surviving flies of Taif. Both Val/Leu + Ala/Val + Tyr and Leu + Ala + Phe/Tyr were prevalent combinations in field populations in Turkey, Japan, and China [4,6,17,48].

Selection pressure is one of the driving forces that stimulates insects to overcome insecticides. The selection pressure affects genes whose products are targets for insecticides [50,51]. In most cases, the pesticide-resistant insect populations depart from Hardy–Weinberg equilibrium [52,53,54]. In this study, the three field house fly populations departed from Hardy–Weinberg equilibrium. Moreover, the three field populations showed little genetic differentiation and high gene flow, suggesting that *Ace* genotypes may move among these populations.

## 5. Conclusions

In conclusion, the house fly field-collected populations from slaughterhouses in Riyadh, Jeddah, and Taif in Saudi Arabia displayed high levels of resistance to PM. Single and combined *Ace* mutations are apparently associated with this resistance. Based on the obtained data, the OP insecticides used in the house fly control programs in Saudi Arabia should be replaced/rotated with insecticides from other insecticide groups, targeting sites other than AChE. The outcomes of the present work can be useful in managing house fly field populations in Saudi Arabia.

## Figures and Tables

**Table 1:** Pirimiphos-methyl median lethal concentration (LC_50_) values and resistance ratios (RR) of *Musca domestica*.

Population	N *	LC_50_ (mM)	95% Confidence Limits		Slope ± SE	χ^2^	*p*	RR
			Lower	Upper				
LAB	360	0.014	0.012	0.017	0.68 ± 0.19	2.32	0.68	-
Riyadh	360	8.44	7.73	9.65	2.63 ± 0.29	2.582	0.28	602.9
Jeddah	360	2.45	2.14	2.74	3.43 ± 0.42	0.755	0.86	175
Taif	360	1.63	1.37	1.96	2.16 ± 0.19	3.04	0.55	116.4

* Number of individual flies.

**Table 2:** Frequency of *Ace* point mutation responsible for MP-resistance in house fly populations, collected from three slaughterhouses in Saudi Arabia.

Population	N *	239 *** ATT→GTT		243 GAG→AAG		260 GTC→CTC		316 GCT→TCT		342 CGC→CC/TC			407 TTT→TAT	
		Ile	Val	Glu	Lys	Val	Leu	Ala	Ser	Gly	Ala	Val	Phe	Tyr
Riyadh	48	98.9	1.1	96.9	3.1	17.7	82.3	100	0	10.4	79.2	10.4	16.7	83.3
Riyadh/S **	12	100	0	100	0	12.5	87.5	100	0	25	70.8	4.2	41.7	58.3
Jeddah	47	97.9	2.1	100	0	23.4	76.6	100	0	13.8	71.3	6.4	23.4	76.6
Jeddah/S **	12	87.5	12.5	100	0	16.7	83.3	100	0	0	83.3	16.7	8.3	91.7
Taif	44	100	0	100	0	27.3	72.7	2.3	97.7	13.6	69.3	13.6	25	75
Taif/S **	9	100	0	100	0	33.3	66.7	100	0	11.1	72.2	16.7	11.1	88.9

* Number of individual flies. ** Survived F1 flies from Riyadh (Riyadh/S), Jeddah (Jeddah/S), and Taif (Taif/S), exposed to different doses of pirimiphos-methyl. *** The mutation positions are underlined and indicated by arrows.

**Table 3:** Frequency of the three *Ace* point mutations, associated with OP resistance, in house fly populations, collected from three slaughterhouses in Saudi Arabia.

Population	N *	Val260Leu GTC/CTC			Gly342Ala/Val ** GGC/GCC/GTC					Phe407Tyr TTT/TAT		
		260			342					407		
		V/V	V/L	L/L	G/G	A/A	G/A	G/V	A/V	F/F	F/Y	Y/Y
Riyadh	48	0	17	31	0	28	10	0	10	3	10	35
		0%	35.4%	64.6%	0%	58.3%	20.8%	0%	20.8%	6.3%	20.8%	72.9%
Jeddah	47	1	20	26	1	25	11	0	6	1	20	26
		2.1%	42.6%	55.3%	2.1%	53.2%	23.4%	0%	12.8%	2.1%	42.6%	55.3%
Taif	44	2	20	22	2	21	7	1	11	3	16	25
		4.5%	45.5%	50%	4.5%	47.7%	15.9%	2.3	25%	6.3%	36.4%	56.8%
KSA	139	3	57	79	3	74	28	1	27	7	46	85
		2%	41%	57%	2%	53%	20%	1%	19%	5%	33.1%	61.9%

* Number of individual flies. ** Individual flies found in Riyadh, Jeddah and Taif; triple alleles at the 342 position were excluded.

**Table 4:** Combinations of three mutations at amino acid positions 260, 342, and 407 of AChE polypeptide, associated with insecticide-resistance, recovered from house flies collected from three slaughterhouses in Saudi Arabia, along with their counterparts, F1 survivors, exposed to pirimiphos-methyl insecticide.

	*Ace* Combination			Population						Total
	260	342	407	Riyadh	Riyadh/S *	Jeddah	Jeddah/S *	Taif	Taif/S *	
1	Leu	Ala	Tyr	22	3	15	6	8	3	57
2	Val/Leu	Ala	Tyr	1	0	0	0	1	1	3
3	Leu	Gly/Ala	Tyr	1	1	1	0	0	0	3
4	Val/Leu	Gly/Ala	Tyr	1	0	3	0	0	0	4
5	Leu	Ala	Phe/Tyr	5	1	9	2	11	0	28
6	Val/Leu	Gly/Ala	Phe/Tyr	5	0	7	0	5	2	19
7	Leu	Ala/Val	Tyr	3	0	0	0	0	3	6
8	Val/Leu	Ala/Val	Tyr	7	1	6	4	11	0	29
9	Val/Leu	Gly/Ala	Phe	3	1	0	0	1	0	4
10	Leu	Gly/Ala	Phe/Tyr	0	3	1	0	1	0	5
11	Leu	Gly/Ala	Phe	0	2	0	0	0	0	2
12	Val	Gly	Phe	0	0	1	0	1	0	2
13	Val/Leu	Gly/Ala/Val	Phe/Tyr	0	0	1	0	1	0	2
14	Val/Leu	Gly/Ala/Val	Tyr	0	0	3	0	1	0	4
15	Val	Gly/Val	Tyr	0	0	0	0	1	0	1
16	Leu	Ala	Phe	0	0	0	0	1	0	1
17	Leu	Gly	Tyr	0	0	0	0	1	0	1
Total				48	12	47	12	44	9	172

* Survived F1 flies from Riyadh (Riyadh/S), Jeddah (Jeddah/S), and Taif (Taif/S), exposed to different doses of pirimiphos-methyl.

**Table 5:** *Ace* genotypes recovered from Saudi field house fly populations and their F_is_ estimates of HWE test.

Recovered *Ace* Genotype	Riyadh	Jeddah	Taif
ACAGCCGTGGGGTGCGTCGAATTCCTCTTAAGTA	8	1	0
ACAGCCGTGGGGTGCGTCRAATYCCTCCTAAGTA	1	0	0
ACAGSYGKGGGGTGYGTYRMATTCCTCTTAARTA	1	0	0
ACAGSYGKGGGGTGYGTYRMATTCCTCTTAAGTA	1	0	0
ACAGCCGTGGGGTGCGTCGAATYCCTCCYAARTA	1	0	0
ACAGSYGKGSGRTGSRTYRMATYCYKCCYAAMTT	1	0	0
ACAGSYGKGGGGTGCGTYRMATYCCTCYYAAGTA	1	0	0
ACAGSYGKGGGGTGYGTYRMATYCCTCYYAAGTA	3	0	0
ACAGCCGTGGGGTGCGTCGAATYCCTCYYAARTA	6	2	2
ACAGCCGTGGGGTGCGTCGAATYCCTCYYAAGTA	1	1	1
ACARCCGTGGGGTGCGTYRMATTCCTCYTAAGTA	1	0	0
ACARCCGTGGGGTGSGTCGAATYCCTCYYAARTA	1	0	0
ACARSCGKGRGGTGSGTYRMATTCCTCYTMARTA	1	0	0
ACAGCCGTGGGGTGCGTCGAATCCCTCCCAARTW	2	2	2
ACAGSCGKGRGGTGSGTYRMATCCCTCCCMAATT	1	0	0
AYAGSCGKRSGGKGSGTYRMATYCCTCCYMARTW	1	0	0
ACAGSCGKGGGGTGSGTCGAATCCCTCCCAAATT	1	0	0
ACAGCCGTGGGGTGCGTCGAATYCCTCYYAARTW	3	4	0
ACAGSCGKGGGGTGYGTYRMATYCCTCYYAAGTA	1	0	0
ACYGSCGKGSGGTGSGTYRMATYCCTCYYAARTW	1	0	0
ACAGCCGTGGGGTGCGTYRMATYCCTCYYAARTA	1	0	0
ACAGSCGKGGGGTGSGTYRMATYCCTCYYMARTW	1	0	0
RCAGCCGTGGGGTGCGTCGAATYCCTCYYAAGTA	1	0	0
ACAGCCGKGGGGTGYGTYRMATYCCTCYYAAGTA	1	0	0
AYAGSCGKRGGGTGSGTYRMATYCCTCYYAAGTW	1	0	0
ACAGSCGKGGGGTGSGTYRMATYCCTCCYAARTW	1	0	0
ACAGSCGKGGGGTGYGTYRMATYCCTCYYAARTA	1	0	0
ACAGCCGTGGGGTGCGTYRMATTCCTCTTAAGTA	1	0	0
ACAGCCGTGGGGTGCGTCGAATCCCTCCCAAGTA	1	0	0
ACAGCCGKGGGGTGYGTYRMATYCCTCYYAARTA	1	0	0
ACAGCYGKGGGGTGYGYYRMATTCCTCTTAARTA	1	0	0
ACAGSCGKGRGGTGSGYTACAYTCCTCTTARGTW	0	1	0
ACAGSYGKGGGGTGBGYYRMATYCCTCYYAARTW	0	1	1
ACAGCCGTGGGGTGCGYYRMATYCCTCYYAARTW	0	3	8
ACAGSCGKGGGGTRSGTCGMATTCYKCWTMAGTW	0	1	0
ACAGSYGKGGGGTGYGYYRMATYCCTCYYAARTW	0	1	0
ACAGCCGTGGGGTGCGYYRMATYCCTCYYAARTA	0	6	2
ACAGSYGKGGGGTGYGYYRMATYCCTCYYAAGTA	0	2	1
ACAGSCGKGGGGTGSGTCGMATYCCTCYYAARTW	0	1	0
ACAGCCGTGGGGTGSGTCGAATCCCTCCCAARTW	0	1	0
ACMGGCRKGGGGTGGGTYRCRTTCCTSCTMAATT	0	1	0
ACAGCCGTGGGGTGCGYYRMATTCCTCYTAARTA	0	1	0
ACAGSCGKGGGGTGSGTYRMMTYCCTSCYAARTW	0	1	0
ACAGSYGKGGGGTGCGYYRMATYCCTCYYAARTW	0	1	0
AYAGSCGKGGGGTGSGYYRMATYMCTCYYAARTW	0	1	0
ACAGSCGKGGGGTGSGYYRMATTCCTCTTAAGTA	0	1	0
ACAGSYGKGGGGTGYGYYRMATYCCTCYYAARTA	0	2	3
ACRGSCGKGSGGTGSGTCGAATTCCTCYTMAGTA	0	1	0
ACAGSYGKGGGGTGSGYYRMATTCCTCYYAARTA	0	1	0
ACAGCCGTGGGGTGSGTCGAATYCCTCYYAARTA	0	1	0
RCAGCCGTGGGGTGCGYYRMATYCCTCYYAAGTA	0	1	0
ACAGSYGKGGGGTGBGYYRMATYCCTCYYAARTA	0	1	0
ACWGSCGKGSGGTGSGTYGAATTCCTCYTAARTW	0	1	0
ACAGCCGTGGGGTGCGYYRMATYCCTCYYAAGTA	0	1	1
RCAGCCGTGGGGTGCGYYRMATTCCTCTTAAGTA	0	1	0
ACAGSYGKGGGGTGBGYYRMATTCCTCTTAARTA	0	1	0
ACAGSYGKGGGGTGYGYYRMATTCCTCTTAAGTA	0	1	3
ACAGSYGKGGGGTGBGYYRMATTCCTCYTAARTA	0	1	0
ACAGCCGTGGGGTGCGTCGAATCCCTCCCAARTA	0	1	1
ACAGSYGKGGGGTGYGYYRMATYCCTCYYARRTA	0	0	1
ACAGSYGKGGGGTGSGYYRMATYCCTCYYAARTA	0	0	1
ACAGSYGGGGKGTGSGYYRMATYCCTCYYAARTA	0	0	1
ACAGSCGKGGGGTGSGTCGMATTCCTGCTAAATT	0	0	1
ACAGSYGKGGGGTGBGCTACATTCCTCTTAAGTA	0	0	1
ACAGSYGKGGKGTGCGYYRMATTCCTCTTAAGTA	0	0	1
ACAGCCGKGGGGTGCGYYRMATYCCTCYYAARTW	0	0	1
ACAGSCGKGGGGTGSGTYRMATYCCTCYTAARTW	0	0	1
ACWGSCGKGGGGTGSGTYRMATYCCTCCYAARTW	0	0	1
ACAGSYGKGGGGTGYGYYRMATTCCTCTTAARTA	0	0	1
ACAGCCGTGGGGTGSGYYRMATYCCTCYYAARTW	0	0	1
ACRGSCGKGGGGTGSGTYRMATTCCTCTTARRTW	0	0	1
ACAGCCGTGGGGTGCGTCGAATTCCTCGTAAGTT	0	0	1
ACWGGCGGGGGGTGGGTTACMTTCCTSCTMGATT	0	0	1
ACGGCCGGGGGGTGGGTTACATTCCTCTTAAACA	0	0	1
ACAGSYGKGGGGTGYGCTACATTCCTCTTAAGTA	0	0	1
AYAGGYGGGSGGTGKGYTACATTCCTCTTAARTA	0	0	1
F_is_ estimates	χ^2^: >59.2703 Df: 34 Prob: <0.004638	χ^2^: >116.3253 Df: 38 Prob: <0.0001	χ^2^: >92.1901 Df: 34 Prob: <0.0001

**Table 6:** Pairwise F_ST_ estimates (below diagonal line) and predicted number of migrants (Nm, above the diagonal line) for the three house fly field populations.

Population	Riyadh	Jeddah	Taif
Riyadh		9	8
Jeddah	0.0171		12
Taif	0.0569	0.0082	

**Table 7:** Nonsynonymous SNPs in *Ace* gene, causing amino acid changes in AChE protein, associated with insecticide resistance in house flies from Saudi Arabia and other countries.

Country	ATA *→ATG	ATT→GTT	GAG→AAG	GTC→CTC	ACG→ATG	GCT→TCT	CGC→CCC/CGC→CTC		TTT→TAT	GGC→GCC	Reference
	Ile162Met **	Ile239Val	Glu243Lys	Val260Leu	Thr310Met	Ala316Ser	Gly342Ala	Gly342Val	Phe470Tyr	Gly445Ala	
KSA	-	+	+	+	-	+	+	+	+	ND	This study
USA	ND	-	-	+	-	+	+	+	+	ND	[5,16]
Japan	+	-	-	+	-	-	+	+	+	ND	[17,48]
Denmark	+	-	-	+	+	-	+	+	+	+	[18]
Kazakhstani	ND	-	-	+	-	-	+	+	+	ND	[35]
Turkey	ND	-	-	+	-	-	+	+	+	ND	[4]
Iran	ND	-	-	-	-	-	+	+	+	ND	NCBI-GenBank, acc# MK257692
China	+	-	-	+	-	-	+	+	+	ND	[34,44]

* The mutation positions are underlined and indicated by arrows. ** The mutation not detected (-), not available (ND) or detected (+).

## Data Availability

The data presented in this study are available from the corresponding author on a reasonable request.
